# The Associations of Caesarean Delivery With Risk of Wheezing Diseases and Changes of T Cells in Children

**DOI:** 10.3389/fimmu.2021.793762

**Published:** 2021-12-14

**Authors:** Jilei Lin, Shuhua Yuan, Bin Dong, Jing Zhang, Lei Zhang, Jinhong Wu, Jiande Chen, Mingyu Tang, Bin Zhang, Hansong Wang, Yuanyuan Dai, Shijian Liu, Yabin Hu, Xinyi Qi, Liangye Xu, Liebin Zhao, Yong Yin

**Affiliations:** ^1^ Department of Respiratory Medicine, Shanghai Children’s Medical Center, Shanghai Jiaotong University School of Medicine, Shanghai, China; ^2^ Pediatric AI Clinical Application and Research Center, Shanghai Children’s Medical Center, Shanghai, China; ^3^ Shanghai Engineering Research Center of Intelligence Pediatrics (SERCIP), Shanghai, China; ^4^ Department of Neonatology, Shanghai Children’s Medical Center, Shanghai Jiaotong University School of Medicine, Shanghai, China; ^5^ Department of Clinical Epidemiology and Biostatistics, Shanghai Children’s Medical Center, School of Medicine, Shanghai Jiao Tong University, Shanghai, China; ^6^ Department of information, Shanghai Children’s Medical Center, Shanghai Jiaotong University School of Medicine, Shanghai, China

**Keywords:** Caesarean delivery, children, asthma, first episode of wheezing, immune cells, age-dependent associations

## Abstract

**Objectives:**

This study aimed to assess the associations of caesarean delivery (CD) with risk of wheezing diseases and changes of immune cells in children.

**Design:**

The cross-sectional study was conducted between May, 2020 and April, 2021.

**Setting and participants:**

The study was conducted in Shanghai Children’s Medical Center, Shanghai, China. A total of 2079 children with a mean age of 36.97 ± 40.27 months and their guardians were included in the present study *via* face-to-face inquiry and physical examination by clinicians.

**Methods:**

Logistic regression was applied to estimate odds ratio (ORs) and 95% confidence intervals (CIs) for the association between CD and first episode of wheezing (FEW) or asthma. Models were adjusted for premature or full-term delivery, exclusive breastfeeding (at least 4 months) or not.

**Results:**

Among the 2079 children, 987 children (47.47%) were born by CD and 1092 (52.53%) by vaginal delivery (VD). Children delivered by caesarean had significantly lower gestational age (P<0.01) compared with those who delivered vaginally. Our results also showed that CD was related to increased risk of FEW by the age of 3(adjusted OR 1.50, 95%CI 1.06, 2.12) and increased tendency to develop asthma by the age of 4 (adjusted OR 3.16, 95%CI 1.25, 9.01). The subgroup analysis revealed that the negative effects of CD on asthma were more obvious in children without exclusive breastfeeding (adjusted OR 4.93, 95%CI 1.53, 21.96) or without postnatal smoking exposure (adjusted OR 3.58, 95%CI 1.20, 13.13). Furthermore, compared with children born through VD, a significant change of the T cells (increased proportion of CD4+ T cells and decreased number and proportion of CD8+ T cells) were observed before the age of one in the CD group. However, the changes were insignificant in children over 1 year old.

**Conclusions:**

This study showed age-dependent associations of CD with asthma and FEW in offspring. Moreover, CD appeared to have an effect on the cellular immunity in infants, the disorder of which may contribute to the development of asthma in children.

## Introduction

In recent decades, the prevalence of caesarean delivery (CD) has risen dramatically in many middle-income and high-income countries. The occurrence of CD has been estimated to be 41.6% in China (1). A large number of pregnant women in China preferred CD (2). Evidence showed that early life environmental exposures of offsprings are important determinants of health and disease in later life (3). Meanwhile, studies have found that CD may have profound effects on the health of offspring (4, 5).

The incidence of the wheezing diseases in children has risen dramatically over past decades (6). Children with wheeze in early life are shown to have a significantly higher risk of developing asthma, which may place a burden on families and society, and lower the life quality of the children (7). Currently, studies have suggested that CD is related to the increased risk of allergic diseases in offspring (8, 9). The potential mechanism may involve that CD interrupts acquisition of mother’s vaginal microbiome. The microbial exposure contributes to intestinal microbiome programming of infants, which can influence immunological development (10, 11). However, there was no clear conclusion about the association between CD and wheeze or asthma (12, 13). The inconsistent results may be related to the heterogeneity of characteristic of different population, including age and races. One previous study showed that there were only minor differences of gut microbiota composition in children with CD compared to children with vaginal delivery (VD) by the age of one (14). Therefore, CD may have age-dependent effects on the offspring. However, there is no study assessing the age-dependent effects of CD on the risk of wheezing diseases and changes of immune cells in children.

Considering factors above, the aim of this study was to assess the age-dependent associations of caesarean delivery with risk of wheezing diseases and changes of immune cells after birth.

## Methods

### Study Design and Participants

The cross-sectional study was conducted in Shanghai Children’s Medical Center, a 1000-bed tertiary teaching hospital in Shanghai, China. Patients who were hospitalized in the department of respiratory medicine were enrolled between May, 2020 and April, 2021. The study was approved by the ethics committee of Shanghai Children’s Medical Center and conducted according to Declaration of Helsinki guidelines.

After being admitted to the department of respiratory medicine, all patients were inquired with detailed information of wheezing diseases, birth status, family history and recorded by clinicians. Flow cytometry of immunocyte was conducted on all the patients. The exclusion criteria were any of the following: 1) the guardians refused to sign the obtaining informed consent, 2) information was seriously absent (the results of wheeze and asthma).

### Data Collection

The collected data included baseline (sex, age, weight, height), birth status (birthday, premature or full-term children, modes of delivery), environmental exposure (breastfeeding status, secondhand smoke exposure), family history (allergic diseases of father, mother and other family members, age of father and mother, work of father and mother), previous wheezing diseases (wheezing in the past, first wheezing time, number of wheezes in the past two years and number of wheezes in the past one year, diagnosis of asthma in the past (see ***Definition***), current status (wheeze, the length of stay in hospital), previous or current other allergic diseases(rhinitis, eczema, food or drug allergy), test of immune cells.

### Definition

Wheeze was defined as record of wheeze reported in the auscultation by pediatricians or general practitioners. The diagnosis of asthma should be either made by the senior attending physician or chief physician or children with a family history of asthma, as well as 3 or more episodes of wheezing (confirmed by pediatrician or general practitioner) and positive pulmonary function (after inhaling β receptor agonist, such as salbutamol, the forced expiratory volume (FEV1) increased ≥ 12%) were diagnosed as having asthma.

### Flow Cytometry and Antibodies

We collected 2 milliliter(mL) peripheral blood from children and placed the samples in EDTA-coated anticoagulant tube. BD Multitest™ 6-color TBNK reagent (https://www.bdbiosciences.com/zh-cn) was used for flow cytometry (FC) of TBNK (T cells, B cells and NK cells, cells/μl). It contains FITC-labeled CD3, clone SK7; PE-labeled CD16, clone B73.1, and CD56, clone NCAM16.2; PerCP-Cy™5.5†–labeled CD45, clone 2D1 (HLe-1); PE-Cy™7–labeled CD4, clone SK3; APC-labeled CD19, clone SJ25C1; and APC-Cy7‡–labeled CD8, clone SK1. For conducting FC of regulatory T cells(Treg), activated T cells, and activated monocytes of peripheral blood, the following antibodies were used: FITC-labeled CD25, PerCP-labeled CD3, APC-labeled CD4, APC-H7-labeled CD4, V450-labeled HLA-DR, V500-labeled CD44 (all were from BD Biosciences), and PE-labeled CD127 (Beekman Coulter).

The procedure of FC for TBNK of peripheral blood: all operations were performed at room temperature (20–25°C). Firstly, we added 20 microliters (ul) of reagent to the BD Trucount™ tubes. Secondly, 50ul blood was added into the reagent. After mixing at low speed, cells were incubated in dark for 15 minutes. Then, 450ul 1xBD FACS lysing solution were added into the system, and mixed at low speed. Incubation in dark for another 15 minutes was then required. Finally, FC was performed on a BD FACSCanto II cytometer. The procedure of FC for Treg, activated T cells, and activated monocytes of peripheral blood: Firstly, the certain amounts of antibodies (according to their instruction) were mixed up with 100ul peripheral blood, then incubated in dark for 15 minutes at room temperature (20–25°C). Secondly, 1ml FAC™ lysing solution (diluted 10 times with deionized water) was added into the system, and incubated in dark for 15 minutes at room temperature (20–25°C). Supernatant was discarded after centrifugation at 1500rpm for 5min. After adding 2ml PBS into the sediment and mixing well, the sample was centrifuged at 1500rpm for 5min and was resuspended by the PBS. Finally, FC was performed on a BD FACSCanto II cytometer.

The absolute number (cells/µL) of positive cells in the sample can be determined by comparing cellular events to bead events. Absolute counts of cells are calculated by BD FACSCanto clinical software using the following formula: cell population absolute count test volume = (events in cell population/events in absolute count bead region)*(beads of each test/test volume).

### Outcomes and Analytic Strategy

Two thousand and seventy-nine patients were divided into two groups according to the delivery mode. We classify all the population by age in the following analysis. Most of the children were preschoolers (**Figure S1**). As for analyses of the first episode of wheezing (FEW), children in 6 groups were extracted from total population according to the age (≥1 years old, ≥2 years old, ≥3 years old, ≥4 years old, ≥5 years old, and ≥6 years old, respectively). As for analyses of asthma, children were extracted from the total population according to the age (≥3 years old, ≥4 years old, ≥5 years old, ≥6 years old, respectively). The age-dependent association between CD and the risk of asthma, FEW and immune cells were estimated. The age-dependent association between CD and FEW or asthma will also be assessed by hazard ratios (HRs), with the development time into FEW and asthma as the underlying time metric. We also estimated the age-dependent effects of CD on the length of stay in hospital (LOS) for wheezing diseases (excluding asthma). The reason why children with asthma were excluded was that in our center, many children with asthma were treated in outpatient clinics or emergency department. Usually, children with asthma admitted into hospital were for the special treatment of omalizumab therapy. Their hospital stay was usually no more than 24 hours. In multivariable analyses, covariate selection was based on the significant difference of factors between the CD and VD groups (P<0.1). Considering that the association of CD with FEW was significant in the first 3 years, we conducted the subgroup analyses in children (≥3 years old) to explore the factors that may affect the effects of delivery modes on FEW in the first 3 years. The association of CD with asthma was significant in the first 4 years, we conducted the subgroup analyses in children above 4 years old to explore the factors that may affect the effect of delivery modes on asthma in the first 4 years.

### Statistical Analysis

In this study, continuous variables were presented as means ± standard deviation (SD). Categorical variables were analyzed by the χ2 test or the Fisher’s exact test, as appropriate. Logistic regression was applied to estimate odds ratio (ORs) and 95% confidence intervals (CIs) for the association between CD and FEW or asthma. The effects of CD on the development of FEW and asthma was assessed by Kaplan–Meier curves and quantified by means of Cox proportional hazards regression (P values correspond to Wald tests). Models were adjusted for premature or full-term delivery and breastfeeding status. Other variables were evaluated but not included in the final model because they were not significant (P>0.1). Prespecified subgroup analyses were conducted to assess whether the observed association of CD and FEW or asthma was affected by sex (girl or boy), father’s allergic diseases (yes or no), mother’s allergic diseases (yes or no), exposure to smoke (yes or no), breastfeeding (exclusive breastfeeding more than 4 months or not), and full-term birth (premature or full-term birth). A P_interaction_ was obtained through a likelihood ratio test, which compares the models with and without interaction terms. P<0.05 was considered to indicate statistical significance. All statistical analyses were performed using SPSS 25.0 software and R 4.1 software.

## Results

### Study Population

We invited the 2079 guardians and their children to participate in the present study. The acquisition method was face-to-face inquiry and conduction of physical examination by clinicians, and the response rate was 100%. The mean age of the children included in our study was 36.97 ± 40.27 months old. Among the 2079 children, 987 children (47.47%) were delivered *via* CD and 1092 children (52.53%) were delivered *via* VD. The descriptive statistics of included children were presented in **Table 1**. At birth, children born by CD had significantly lower gestational age (P<0.01). There was no significant difference of family history of allergic diseases between CD and VD groups. In this study, the difference of the incidences of FEW, recurrent wheeze and asthma was insignificant between CD and VD groups. And the difference of the time for developing into FEW and asthma was also insignificant between CD and VD groups. Like the above results, there was no significant difference of immune cells numbers between CD and VD groups.

**Table 1:** The clinical characteristics of included children.

	Total		CD		VD		P
	N	Values	N	Values	N	Values	
**Birth**							
Sex [boy, %]	2079	1182 (56.85%)	987	568 (57.55%)	1092	614 (56.23%)	0.54
Age [mean ± sd, months old]	2079	36.97 ± 40.27	987	36.5 ± 41.11	1092	37.4 ± 39.5	0.61
Premature delivery [yes, %]	2079	222 (10.68%)	987	157 (15.91%)	1092	65 (5.95%)	**<0.01**
Father’s allergic diseases [yes, %]	2079	384 (18.47%)	987	189 (19.15%)	1092	195 (17.86%)	0.45
Maternal allergic diseases [yes, %]	2079	294 (14.14%)	987	131 (13.27%)	1092	163 (14.93%)	0.28
Other family members’ allergic diseases [yes, %]	2079	149 (7.17%)	987	72 (7.29%)	1092	77 (7.05%)	0.83
**Postnatal exposure**							
Exclusive breastfeeding [yes, %]	2079	658 (31.65%)	987	267 (27.05%)	1092	391 (35.81%)	**<0.01**
Exposure to smoke [yes, %]	2079	391(118.81%)	987	193 (19.55%)	1092	198 (18.13%)	0.41
**Outcomes**							
First episode of wheezing [yes, %]	2079	724 (34.82%)	987	367 (37.18%)	1092	357 (32.69%)	0.03
Length of time for development of FTW [mean ± sd, months]	724	18.33 ± 20.34	367	17.78 ± 20.33	357	18.90 ± 20.37	0.46
Recurrent wheeze [yes, %]	2079	417(20.06%)	987	207 (20.97%)	1092	210 (19.23%)	0.32
Asthma [yes, %]	2076	267 (12.84%)	985	146 (14.82%)	1091	121 (11.09%)	0.01
Length of time for development of asthma [mean ± sd, months]	267	48.35 ± 31.45	146	48.47 ± 32.95	121	48.20 ± 29.67	0.94
LOS [mean ± sd, day]	2079	6.60 ± 6.40	987	6.35 ± 5.43	1092	6.8 ± 7.09	0.1
LOS for wheezing diseases (not asthma) [mean ± sd, day]	503	6.01 ± 4.71	347	6.03 ± 4.86	256	5.97 ± 4.47	0.93
**Test of immune cells**							
CD3+	1697	3418.35 ± 2868.99	807	3343.25 ± 2725.54	890	3486.45 ± 2993.02	0.3
CD3+%	1697	63.62 ± 11.67	807	63.09 ± 11.73	890	64.09 ± 11.59	0.08
CD3+CD4+	1697	1823.26 ± 1258.3	807	1816.42 ± 1245.17	890	1829.46 ± 1270.76	0.83
CD3+CD4+%	1697	35.55 ± 10.79	807	35.72 ± 11.01	890	35.39 ± 10.59	0.54
CD3+CD8+	1697	1368.33 ± 2107.98	807	1305.32 ± 1939.54	890	1425.46 ± 2249.41	0.24
CD3+CD8+%	1697	23.25 ± 11.9	807	22.62 ± 11.69	890	23.83 ± 12.07	0.04
CD4/CD8	1697	1.93 ± 1.21	807	2.01 ± 1.33	890	1.86 ± 1.07	0.01
CD3+/HLA-DR+	1388	590.98 ± 1864.55	664	582.02 ± 1796.89	724	599.19 ± 1925.72	0.86
CD3+/HLA-DR+%	1404	8.2 ± 21.64	669	7.7 ± 12.41	735	8.65 ± 27.47	0.4
Treg	1421	129.4 ± 108.52	677	133.02 ± 117.85	744	126.11 ± 99.24	0.23
Treg%	1440	3.56 ± 18.92	683	3.59 ± 16.07	757	3.54 ± 21.18	0.96
CD3-/CD19+	1697	1135.12 ± 876.03	807	1119.8 ± 853.2	890	1149.02 ± 896.47	0.49
CD3-/CD19+%	1697	23.05 ± 10.61	807	23.39 ± 10.89	890	22.74 ± 10.34	0.21
CD45+	1697	5231.69 ± 3734.08	807	5112.76 ± 3529.76	890	5339.53 ± 3908.98	0.21
NK	1697	618.8 ± 589.15	807	600.75 ± 517.67	890	635.17 ± 647.03	0.22
NK%	1697	12.03 ± 7.42	807	12.14 ± 7.26	890	11.93 ± 7.55	0.55
CD14+/HLA-DR+	1389	898.21 ± 1476.54	665	848.34 ± 659.08	724	944.01 ± 1944.77	0.21
CD14+/HLA-DR+%	1408	84.94 ± 29.44	671	84.12 ± 23.05	737	85.67 ± 34.24	0.32

CD, caesarean delivery; VD, vaginal delivery; sd, standard deviation; N, Number of sample size.CD3+, The number of CD3+ T cells/ul; CD3+%, The ratio of CD3+ T cell in total lymphocyte; CD3+CD4+, The number of CD3+CD4+ T cells/ul; CD3+CD4+%, The ratio of CD3+CD4+ T cell in total lymphocyte; CD3+CD8+, The number of CD3+CD8+ T cells/ul; CD3+CD8+%, The ratio of CD3+CD8+ T cell in total lymphocyte; CD4/CD8, the ratio of CD4+ T cell versus CD8+ T cell; CD3+/HLA-DR+, The number of CD3+/HLA-DR+ T cells/ul; CD3+/HLA-DR+%, The ratio of CD3+/HLA-DR+ T cell in total lymphocyte; Treg, The number of regulatory T cells/ul; Treg%, The ratio of regulatory T cell in total lymphocyte; CD3-/CD19+, The number of B cells/ul; CD3-/CD19+%, The ratio of B cell in total lymphocyte; CD45+, The number of CD45+ cells/ul; NK, The number of NK cells/ul; NK%, The ratio of NK cell in total lymphocyte; CD14+/HLA-DR+, The number of activated monocytes cells/ul; CD14+/HLA-DR+%, The ratio of activated monocytes in total monocytes.

All tests of these cells are done by flow cytometry. The absolute number (cells/µL) of positive cells in the sample can be determined by comparing cellular events to bead events. Absolute counts of cells are calculated by BD FACSCanto clinical software using the following formula: cell population absolute count test volume = (events in cell population/events in absolute count bead region)*(beads of each test/test volume).

In bold <0.01: P values less than 0.01.

### The Associations of Delivery Mode With First Episode of Wheezing

**Figure 1:**
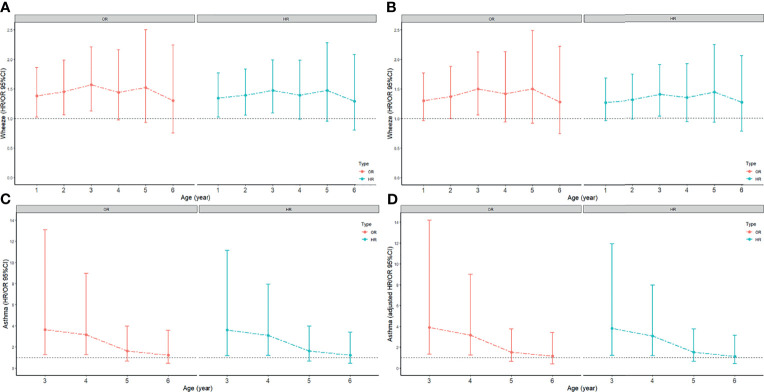
The odds ratio (ORs) and 95% confidence intervals (CIs) for the association between CD and FEW or asthma. Models were adjusted for premature or full-term delivery, breastfeeding status. The age-dependent association between CD and FEW or asthma was assessed by hazard ratios (HRs), with time of development of FEW and asthma as the underlying time metric. **(A)** the crude ORs and HRs for FEW; **(B)** the adjusted ORs and adjusted HRs for FEW; **(C)** the crude ORs and HRs for asthma; **(D)** the adjusted ORs and adjusted HRs for asthma.

All data of FEW were valid. Children were divided into 6 groups by age (≥1 years old, ≥2 years old, ≥3 years old, ≥4 years old, ≥5 years old, ≥6 years old). Each group had 1373, 1035, 800, 540, 374, 302 children respectively. The risk of FEW in the first 1, 2, 3, 4, 5, and 6 years after birth were evaluated. Univariate and multivariate logistic regression analyses suggested that CD increased the risk of FEW in the first 3 years (adjusted OR 1.50, 95%CI 1.06 to 2.12) (**Figure 1**). Cox proportional hazards regression analyses suggested that CD reduced the time for developing into FEW in the first 3 years (adjusted HR 1.4, 95%CI 1.03 to 1.90) (**Figure 1**). The detailed information of the ORs and HRs were shown in **Table S1**.

Subgroup analyses were conducted to explore the potential influencing factors for the effects of delivery mode in the first 3 years. The detailed information of the subgroup analyses was shown in **Table 2**. CD was found to be associated with higher risks of FEW in girls (adjusted OR 2.04, 95% CI, 1.18 to 3.55), in children who had a father without allergic diseases (adjusted OR 1.54; 95% CI, 1.01 to 2.33), in children who had a mother without allergic diseases (adjusted OR 1.79, 95% CI, 1.21 to 2.67), in children without smoke exposure (adjusted OR 1.49; 95% CI, 1.02 to 2.2), in children without exclusive breastfeeding (adjusted OR 1.78, 95% CI, 1.15 to 2.76), and in full-term children (adjusted OR 1.59; 95% CI, 1.10 to 2.29). However, there was no significant interaction between CD and above factors for FEW (all P_interaction_ > 0.05).

**Table 2:** Subgroup analyses on the association between caesarean delivery and asthma or first time of wheeze.

	N	Crude OR (95%CI)	P_interaction_	Adjusted OR (95%CI)	P_interaction_
**Risk of first episode of wheezing in first 3 years**					
Sex					
Girl	354	2.16 (1.28, 3.68)	0.11	2.04 (1.18, 3.55)	0.14
Boy	446	1.23 (0.78, 1.92)		1.19 (0.76, 1.87)	
Father’s allergic diseases					
No	637	1.59 (1.06, 2.39)	0.89	1.54 (1.01, 2.33)	0.99
Yes	163	1.5 (0.78, 2.9)		1.46 (0.75, 2.85)	
Maternal allergic diseases					
No	686	1.88 (1.28, 2.78)	0.08	1.79 (1.21, 2.67)	0.11
Yes	114	0.86 (0.39, 1.86)		0.73 (0.31, 1.66)	
Exposure to smoke					
No	649	1.57 (1.08, 2.3)	0.98	1.49 (1.02, 2.2)	0.98
Yes	151	1.55 (0.72, 3.44)		1.53 (0.7, 3.44)	
Exclusive breastfeeding					
No	534	1.87 (1.22, 2.9)	0.21	1.78 (1.15, 2.76)	0.21
Yes	266	1.18 (0.67, 2.09)		1.11 (0.62, 1.98)	
Premature delivery					
No	737	1.57 (1.09, 2.26)	0.34	1.59 (1.1, 2.29)	0.32
Yes	63	0.9 (0.31, 2.73)		0.89 (0.3, 2.7)	
					
**Risk of Asthma in first 4 years**					
Sex					
Girl	230	2.57 (0.86, 8.60)	0.42	2.27 (0.74, 7.72)	0.42
Boy	307	6.95 (1.17, 132.10)		7.04 (1.18, 134.21)	
Father’s allergic diseases					
No	434	2.91 (1.04, 9.37)	0.78	2.77 (0.98, 9.01)	0.77
Yes	103	4.17 (0.59, 83.08)		5.33 (0.71, 109.89)	
Maternal allergic diseases					
No	459	3.78 (1.29, 13.69)	0.54	3.85 (1.31, 13.97)	0.54
Yes	78	1.92 (0.30, 15.25)		1.61 (0.23, 13.63)	
Exposure to smoke					
No	426	3.53 (1.19, 12.9)	0.71	3.58 (1.20, 13.13)	0.71
Yes	111	2.37 (0.44, 17.67)		2.2 (0.40, 16.53)	
Exclusive breastfeeding					
No	378	4.80 (1.49, 21.33)	0.25	4.93 (1.53, 21.96)	0.25
Yes	159	1.43 (0.26, 7.94)		1.37 (0.24, 7.71)	
Premature delivery					
No	498	3.74 (1.41, 11.73)	0.24	3.72 (1.40, 11.67)	0.24
Yes	39	0.61 (0.02, 16.22)		0.73 (0.03, 21.34)	

N, Number of sample size; Adjusted, Models were adjusted for premature or full-term delivery and breastfeeding status.

### The Associations of Delivery Mode With Asthma

First of all, twelve children without clear information for asthma were omitted. And children were divided into 4 groups by age (≥3 years old, ≥4 years old, ≥5 years old, ≥6 years old). Each group had 797, 537, 371, 299 children respectively. The risk of asthma in the first 3, 4, 5, and 6 years after birth was evaluated. Univariate and multivariate logistic regression analyses between asthma and delivery mode suggested that CD increased the risk of asthma in the first 4 years (adjusted OR 3.16, 95%CI 1.25 to 9.01) (**Figure 1**). Cox proportional hazards regression analyses between asthma and delivery mode suggested that CD reduced the time for developing into asthma in the first 4 years (adjusted HR 3.07, 95%CI 1.19 to 7.95) (**Figure 1**). The detailed information of the ORs and HRs were shown in **Table S1**.

Subgroup analyses were conducted to explore the potential influencing factors for the effects of delivery mode on the risk of asthma in the first 4 years. CD was found to be associated with higher risks of asthma in boys (adjusted OR 7.04; 95% CI, 1.18-134.21), in children who had a mother without allergic diseases (adjusted OR 3.85; 95% CI, 1.31 to 13.97), in children without smoke exposure (adjusted OR 3.58; 95% CI, 1.20-13.13), in children without exclusive breastfeeding (adjusted OR 4.93; 95% CI, 1.53 to 21.96), and in full-term children (adjusted OR 3.72; 95% CI, 1.40 to 11.67). However, there was no significant interaction between CD and above factors for asthma (all P_interaction_> 0.05). The detailed information of the subgroup analyses was shown in **Table 2**.

### The Associations of Delivery Mode With LOS of Wheezing Diseases

**Figure 2:**
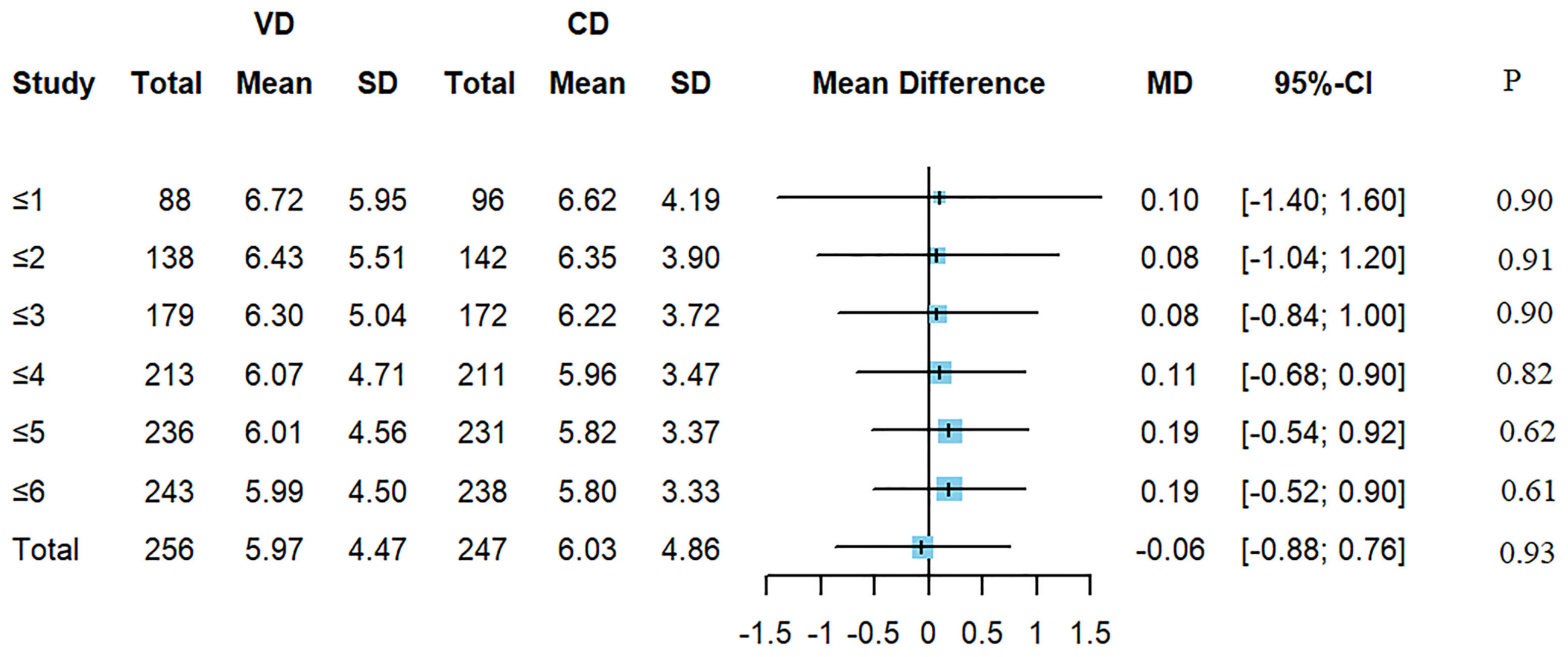
The mean difference of LOS of wheezing diseases between CD and VD groups.

There were 503 children with wheeze (excluding asthma) during this hospitalization. Children were divided into 6 groups by age (≤1 years old, ≤2 years old, ≤3 years old, ≤4 years old, ≤5 years old, ≤6 years old). Each group had 184, 280, 351, 424, 467, 481 children respectively. **Figure 2** suggested that there was no difference of the LOS of wheezing diseases between CD and VD groups (**Figure 2**).

### The Associations of Delivery Mode With Levels of Immune Cells

About 1697 guardians approved to conduct the flow cytometry (FC) for immune cells, however, a few people refused to complete the FC. The results of cytology were shown in **Table 1**. There was no significant difference of immune cells between CD and VD groups. To explore whether CD had different effects on immune cells at different ages, we divided these children according to age (≤1 years old, 1~2 years old, 2~3 years old, 3~4 years old, 4~5 years old, 5~6 years old). The results suggested that compared with the VD group, CD4+ T cells were increased in CD group before the age of 1 (P<0.01), and CD8+ T cells were decreased in CD group before the age of 1 (P<0.01). The results of CD4+ and CD8+ T cells were shown in **Table 3**. The related plots of FC of children under 1 years old were shown in **Figure S4**. And the detailed information of all immune cells was shown in **Table S2**.

**Table 3:** The changes of CD4+ or CD8+ T cells between VD and CD group in different age.

Age (years)	CD4+ and/or CD8+ T cells	VD		CD		P
		N	Values	N	Values	
<=1	CD3+CD4+	285	2709.55 ± 1448.23	285	2730.23 ± 1229.15	0.85
	CD3+CD4+%	285	39.44 ± 9.99	285	42.32 ± 10.4	**<0.01**
	CD3+CD8+	285	1411.55 ± 1120.13	285	1311.94 ± 1795.2	0.43
	CD3+CD8+%	285	19.88 ± 7.64	285	18.31 ± 7.48	**0.01**
	CD4/CD8	285	2.35 ± 1.24	285	2.78 ± 1.71	**<0.01**
1~2	CD3+CD4+	149	2029.22 ± 1230.33	132	1777.88 ± 1159.99	0.08
	CD3+CD4+%	149	35.29 ± 10.04	132	32.98 ± 9.93	**0.05**
	CD3+CD8+	149	1230.47 ± 1028.16	132	1423.72 ± 1818.7	0.28
	CD3+CD8+%	149	20.85 ± 8.45	132	21.91 ± 10.44	0.35
	CD4/CD8	149	1.99 ± 1.05	132	1.85 ± 1	0.25
2~3	CD3+CD4+	111	1546.62 ± 795.08	86	1620.31 ± 1019.01	0.58
	CD3+CD4+%	111	33.38 ± 10.47	86	32.57 ± 9.61	0.58
	CD3+CD8+	111	1618.94 ± 3026.34	86	1512.16 ± 2110.83	0.77
	CD3+CD8+%	111	24.65 ± 13.66	86	23.96 ± 13.6	0.73
	CD4/CD8	111	1.73 ± 0.95	86	1.7 ± 0.86	0.82
3~4	CD3+CD4+	114	1308.25 ± 657.97	106	1140.97 ± 676.21	0.06
	CD3+CD4+%	114	33.33 ± 9.99	106	32.23 ± 8.65	0.39
	CD3+CD8+	114	1640.97 ± 3843.35	106	1098.22 ± 1865.17	0.18
	CD3+CD8+%	114	26.19 ± 13.69	106	23.55 ± 12.04	0.13
	CD4/CD8	114	1.56 ± 0.71	106	1.64 ± 0.73	0.39
4~5	CD3+CD4+	94	1098.02 ± 610.15	54	1183.16 ± 676.25	0.45
	CD3+CD4+%	94	33.13 ± 9.58	54	32.77 ± 7.47	0.8
	CD3+CD8+	94	1144.09 ± 1900.14	54	1191.64 ± 1865.78	0.88
	CD3+CD8+%	94	25.35 ± 12.12	54	25.8 ± 10.53	0.82
	CD4/CD8	94	1.56 ± 0.92	54	1.45 ± 0.57	0.37
5~6	CD3+CD4+	32	1044.18 ± 575.18	30	1051.5 ± 777.02	0.97
	CD3+CD4+%	32	30.47 ± 9.66	30	32.88 ± 10.35	0.35
	CD3+CD8+	32	1544.19 ± 2633.29	30	1046.16 ± 1942.18	0.4
	CD3+CD8+%	32	30.27 ± 14.39	30	24.22 ± 12.2	0.08
	CD4/CD8	32	1.18 ± 0.51	30	1.57 ± 0.67	0.01

CD, caesarean delivery; VD, vaginal delivery; N, Number of sample size; CD3+, The number of CD3+ T cells/μl; CD3+%, The ratio of CD3+ T cell in total lymphocyte; CD3+CD4+, The number of CD3+CD4+ T cells/μl; CD3+CD4+%, The ratio of CD3+CD4+ T cell in total lymphocyte; CD3+CD8+, The number of CD3+CD8+ T cells/μl; CD3+CD8+%, The ratio of CD3+CD8+ T cell in total lymphocyte; CD4/CD8, the ratio of CD4+ T cell versus CD8+ T cell; CD3+/HLA-DR+, The number of CD3+/HLA-DR+ T cells/μl; CD3+/HLA-DR+%, The ratio of CD3+/HLA-DR+ T cell in total lymphocyte; Treg, The number of regulatory T cells/μl; Treg%, The ratio of regulatory T cell in total lymphocyte; CD3-/CD19+, The number of B cells/μl; CD3-/CD19+%, The ratio of B cell in total lymphocyte; CD45+, The number of CD45+ cells/μl; NK, The number of NK cells/μl; NK%, The ratio of NK cell in total lymphocyte; CD14+/HLA-DR+, The number of activated monocytes cells/μl; CD14+/HLA-DR+%, The ratio of activated monocytes in total monocytes. All tests of these cells are done by flow cytometry. The absolute number (cells/µL) of positive cells in the sample can be determined by comparing cellular events to bead events. Absolute counts of cells are calculated by BD FACSCanto clinical software using the following formula: cell population absolute count test volume = (events in cell population/events in absolute count bead region)*(beads of each test/ test volume).

The bold values meant the significant difference between two groups.

## Discussion

In this observational study, we revealed significant age-dependent associations of CD with the risk of asthma and FEW. Subgroups analyses further showed that these associations might be different in different age group. No significant age-dependent association was observed for LOS for wheezing diseases. Significant difference of CD4+ and CD8+ T cells in early life (before the age of 1) was found between CD and VD groups.

Several studies had examined the association of CD with asthma (8, 14, 15), but no study focused on age-dependent effects of CD on asthma or wheeze. In this study, we conducted physical examination, medical history inquiry and questionnaire survey on each child hospitalized in the respiratory department. Five hundred and three children in the hospital had the symptoms of wheezing. We inquire guardians about the past medical history of their children, and looked through children’s medical records. We combined the children with medical history of wheeze and the children with current wheeze as the final data. In this study, we revealed the association of CD with the risk of asthma and FEW in early life, but this association gradually became insignificant as the age passes 4 years old. Our results suggested that the inconsistency of previous cross-sectional studies may result from the age difference of the different study population. Of note, one previous study documented a group of eight-year-old children (40 in India and 155 in Vietnam12) and their result showed a connection between CD and asthma. The small number of participants in their study may lead to limited reliability of the conclusion. The inconsistency could also be due to the differences in sociodemographic characteristics.

Interestingly, the results of subgroup analysis showed that CD was associated with higher risks of asthma in some specific population, such as boys, children without smoke exposure, and children without exclusive breastfeeding. However, the P for interaction was insignificant among them, which indicated that these factors might not truly modify the effects of CD on asthma or FEW. However, some P values were close to 0.05, suggesting that we need to further find evidence from more population.

Our results showed that CD may affect the cellular immunity in infants and the profiles of the T cells. Increased proportion of CD4+ T cells and decreased number and proportion of CD8+ T cells were observed in CD group. The T cells in those less than one is not in the same children that then go on to develop wheeze later. One previous study showed a similar results that pre-labor cesarean section might contribute to the increasing ratio of CD4+ T cells (16). One previous study showed similar changes of CD4+ and CD8+ T cells in cord blood of mother with air pollutants during pregnancy (17). Therefore, it is necessary to further explore the mechanism of the effects of CD on T cells, which may help to reveal the mechanism of the effect of CD on allergic diseases. It is noteworthy that according to the results of our subgroup analysis, the risk of asthma or wheeze in children with CD may be reduced by increasing exclusive breastfeeding. What is more, one previous study showed that the differences of gut microbiota composition between children with CD and children with VD was significant before the age of 1 (14). The intestinal microbiome programming of infants influences immunological development. Interestingly, our study also found that the difference of immune cells before the age of 1 was significant between CD and VD groups. It deserves further study if immune cells, together with gut microbiota composition contribute to the association between CD and wheezing diseases before the age of one.

This study had a number of strengths. This is the first study to explore the age-dependent associations of CD with risk of asthma and FEW. Furthermore, our study found that the changes of CD4+ T cells and CD8+ T cells in the early life of offsprings may be the potential mechanism for the age-dependent association of CD with asthma.

Our study has several limitations. Firstly. We divided all of the participants by age, however, we did not observe the same group of people at different age, which may lead to bias. Secondly, since this is a retrospective cohort study, recall bias might occur when the medical history was collected from the guardians. However, this bias is nondifferential, because it was not expected to be associated with future asthma. Furthermore, though we adjusted for several confounders, our results may still be subject to residual confounding due to unrecognized or unmeasured confounders.

## Conclusions

The results of our study suggested that CD is associated with increased risks of FEW only in the first 3 years and asthma in the first 4 years. However, these findings need to be confirmed in other populations and settings. The significant differences of CD4+ and CD8+ T cells in infants were found between CD and VD groups, which suggested future studies could focus on the role of CD4+ or CD8+ T cells in CD associated wheeze or asthma in early life.

## Data Availability Statement

The raw data supporting the conclusions of this article will be made available by the authors, without undue reservation.

## Ethics Statement

The study design was approved by the ethics committee of Shanghai Children’s Medical Center, Shanghai Jiaotong University School of Medicine and conducted according to the Declaration of Helsinki guidelines (SCMCIRB-W2021055). Written informed consent to participate in this study was provided by the participants’ legal guardian/next of kin.

## Author Contributions

JL, SY and BD conceptualized and designed the study, supervised data collection, carried out the initial analyses, drafted the initial manuscript. JZ, LZ and JW designed the data collection instruments and collected data. JC, MT, BZ, HW, YD and XQ coordinated and supervised data collection, assisted in the statistical analysis and carried out the initial analyses. SL and YH coordinated and supervised data collection, and critically reviewed the manuscript for important intellectual content. YY, LBZ and LX conceptualized and designed the study, supervised data collection, reviewed and revised the manuscript. All authors read and approved the final manuscript.

## Funding

This work was supported by the Science and Technology Innovation-Biomedical Supporting Program of Shanghai Science and Technology Committee [No.19441904400]; the Science and Technology Innovation-Biomedical Supporting Program of Shanghai Science and Technology Committee [No.19441909000]; Program for artificial intelligence innovation and development of Shanghai Municipal Commission of Economy and Informatization [No.2020-RGZN-02048].

## Conflict of Interest

The authors declare that the research was conducted in the absence of any commercial or financial relationships that could be construed as a potential conflict of interest.

## Publisher’s Note

All claims expressed in this article are solely those of the authors and do not necessarily represent those of their affiliated organizations, or those of the publisher, the editors and the reviewers. Any product that may be evaluated in this article, or claim that may be made by its manufacturer, is not guaranteed or endorsed by the publisher.
