# Supplementary material for: The Associations of Caesarean Delivery With Risk of Wheezing Diseases and Changes of T Cells in Children
Source: Front Immunol. 2021 Dec 14;12:793762. doi: 10.3389/fimmu.2021.793762 (PMC8712489; doi:10.3389/fimmu.2021.793762)
Supplement: Supplementary file 1 [file DataSheet_1.pdf]

Table S1: the detailed information about the estimate effects of caesarean delivery on asthma or first episode of wheezing

	Time point (years)	N	Crude OR (95%CI)	Adjusted OR (95%CI)	Crude HR (95%CI)	Adjusted HR (95%CI)
First episode of wheezing	0~1	1373	**1.38 (1.02, 1.86)**	1.3 (0.96, 1.77)	**1.34 (1.02, 1.77)**	1.27 (0.96, 1.68)
	0~2	1035	**1.45 (1.06, 1.98)**	**1.37 (1.01, 1.88)**	**1.39 (1.05, 1.83)**	1.31 (0.98, 1.74)
	0~3	800	**1.57 (1.12, 2.21)**	**1.5 (1.06, 2.12)**	**1.47 (1.09, 1.99)**	**1.4 (1.03, 1.90)**
	0~4	540	1.44 (0.97, 2.16)	1.42 (0.94, 2.13)	1.39 (0.98, 1.98)	1.35 (0.94, 1.92)
	0~5	374	1.52 (0.93, 2.5)	1.5 (0.92, 2.49)	1.47 (0.95, 2.28)	1.44 (0.93, 2.24)
	0~6	302	1.3 (0.75, 2.24)	1.28 (0.74, 2.22)	1.29 (0.8, 2.08)	1.27 (0.78, 2.05)
Asthma	0~3	797	**3.64 (1.25, 13.1)**	**3.9 (1.33, 14.18)**	**3.59 (1.16, 11.14)**	**3.82 (1.22, 11.94)**
	0~4	537	**3.16 (1.26, 8.97)**	**3.16 (1.25, 9.01)**	**3.08 (1.20, 7.95)**	**3.07 (1.19, 7.95)**
	0~5	371	1.62 (0.66, 3.96)	1.52 (0.62, 3.75)	1.62 (0.66, 3.96)	1.52 (0.62, 3.75)
	0~6	299	1.23 (0.43, 3.58)	1.15 (0.39, 3.42)	1.23 (0.45, 3.39)	1.13 (0.41, 3.14)

N: Number of sample size; OR: Odds ratio; HR: hazard ratios;

Adjusted: Models were adjusted for premature or full-term delivery and breastfeeding status.

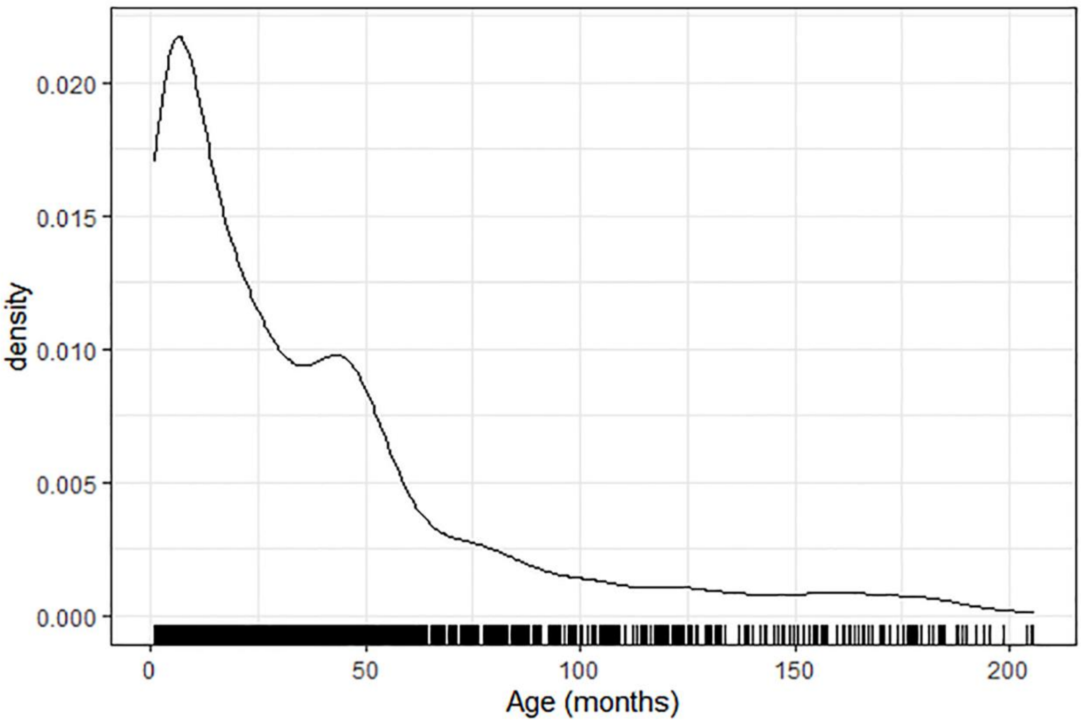

Figure S1: the distribution of age of all children.

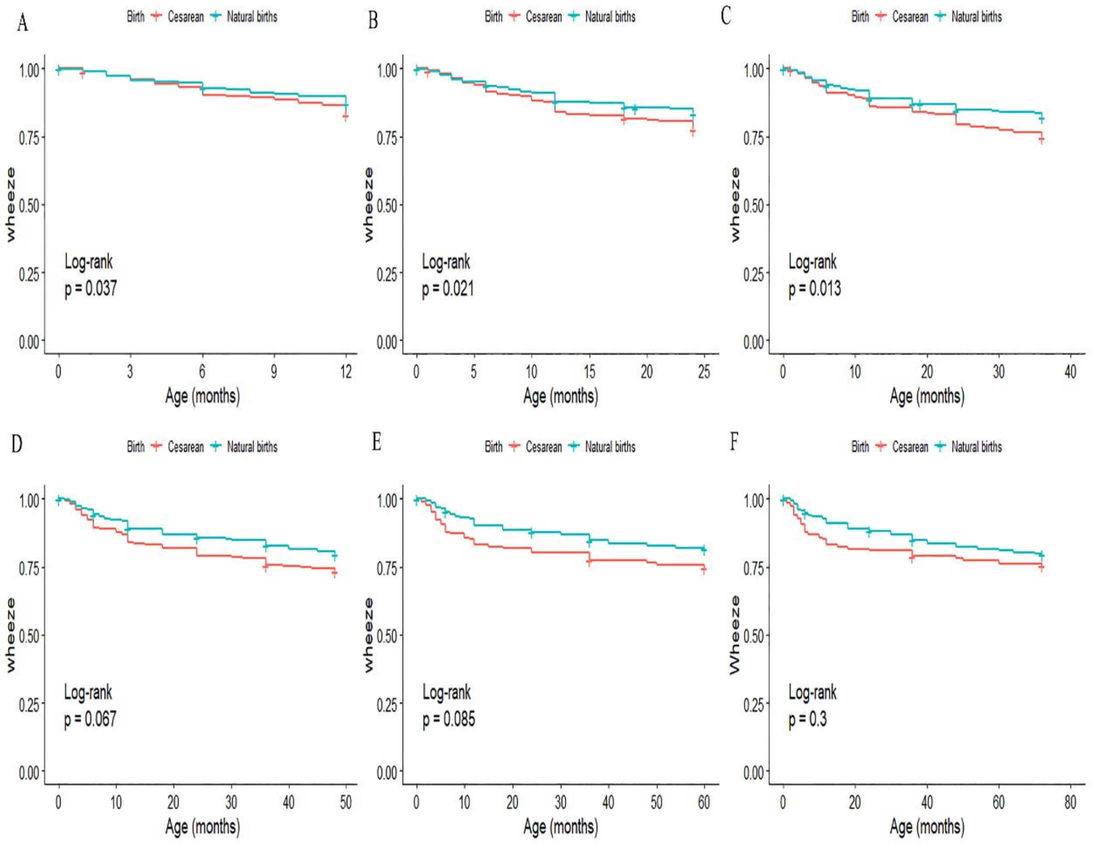

Figure S2: The effects on the development of FEW was assessed by Kaplan–Meier curves and quantified by means of Cox proportional hazards regression (P values correspond to Wald tests). A: in first 1 year; B: in first 2 year; C: in first 3 year; D: in first 4 year; E: in first 5 year; F in first 6 year.

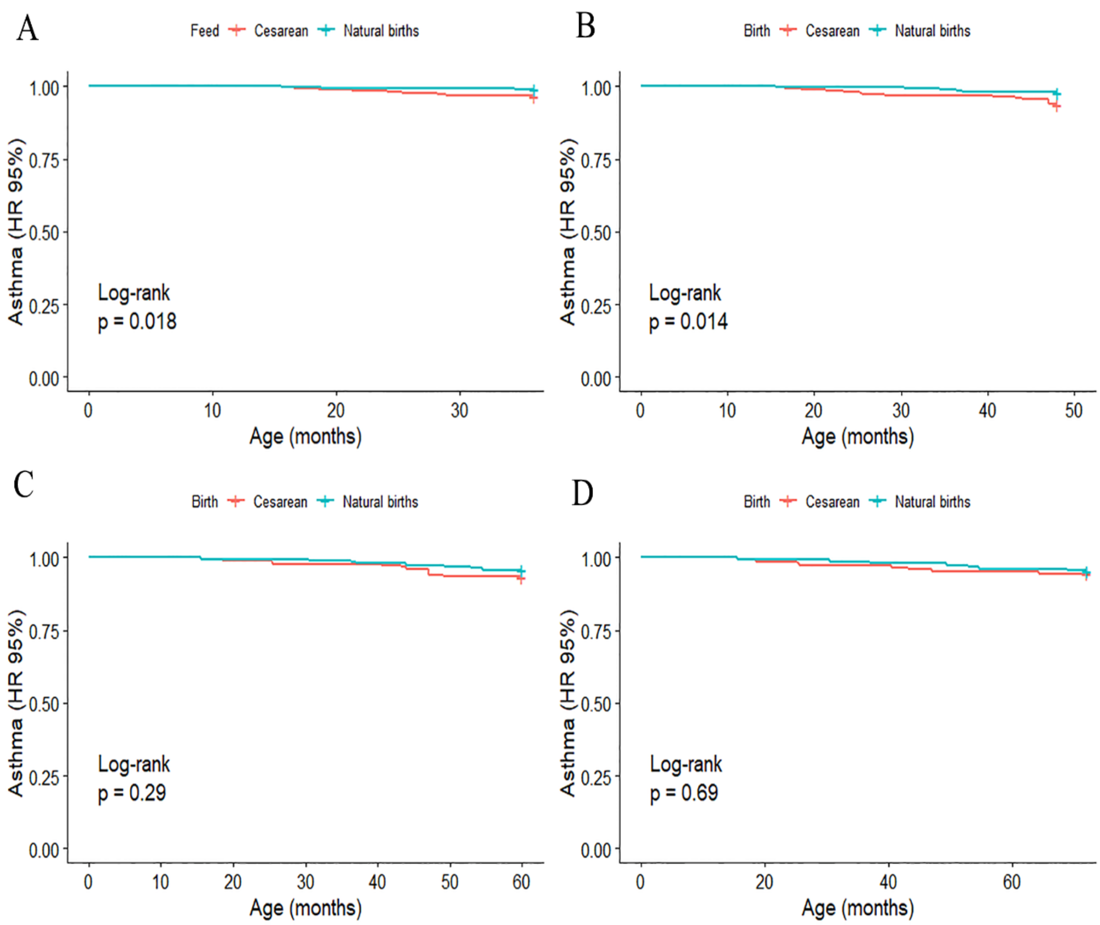

Figure S3: The effects on the development of asthma was assessed by Kaplan–Meier curves and quantified by means of Cox proportional hazards regression (P values correspond to Wald tests). A: in first 3 year; B: in first 4 year; C: in first 5 year; D: in first 6 year.

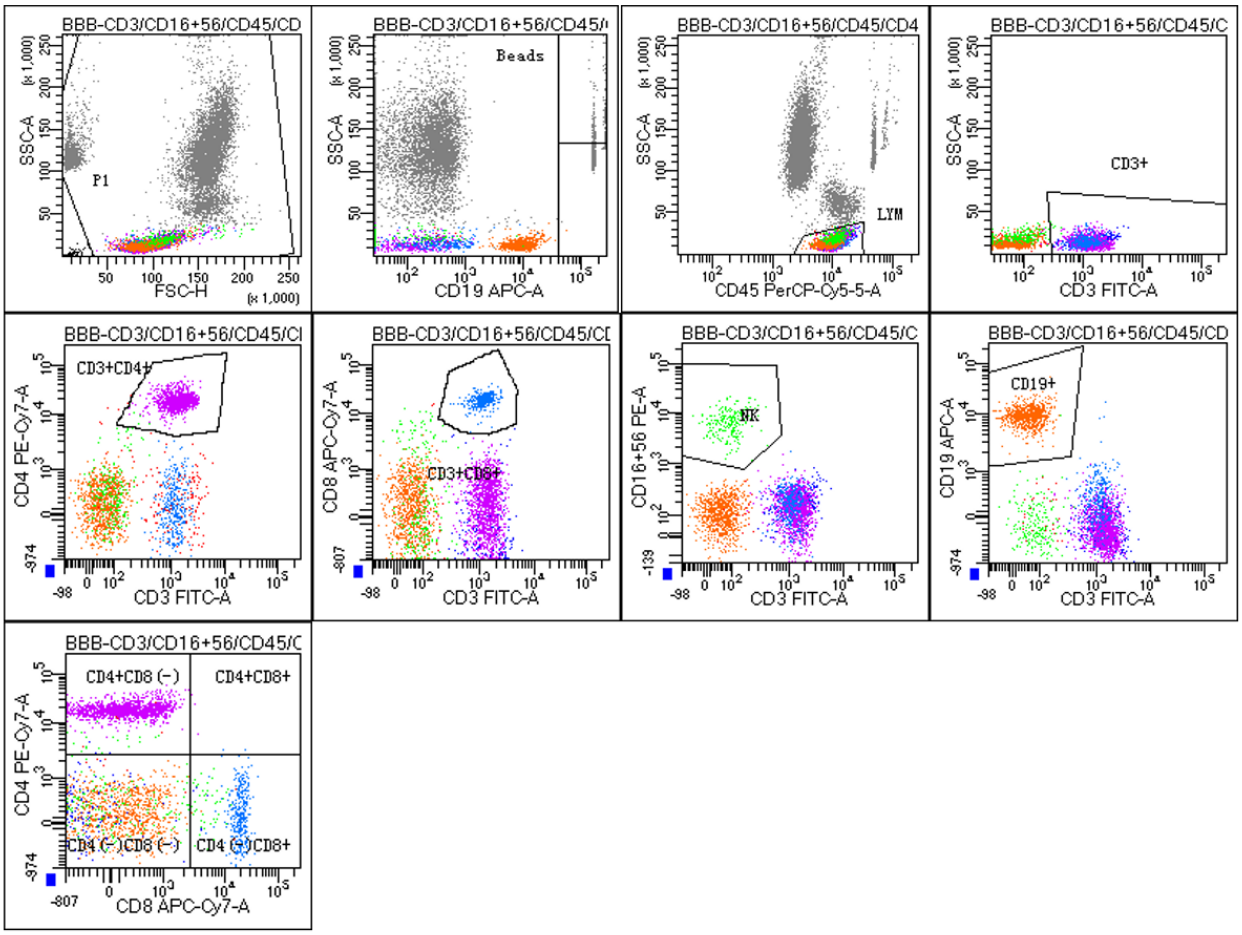

Figure S4: The flow cytometry of immune cells in children. BD Multitest™ 6-color TBNK reagent (https://www.bdbiosciences.com/zh-cn) was used for flow cytometry (FC) of TBNK (T cells, B cells and NK cells, cells/μl). It contains FITC-labeled CD3, clone SK7; PE-labeled CD16, clone B73.1, and CD56, clone NCAM16.2; PerCP-Cy™5.5†–labeled CD45, clone 2D1 (HLe-1); PE-Cy™7–labeled CD4, clone SK3; APC-labeled CD19, clone SJ25C1; and APC-Cy7‡–labeled CD8, clone SK1. The absolute number (cells/μL) of positive cells in the sample can be determined by comparing cellular events to bead events. Absolute counts of cells are calculated by BD FACSCanto clinical software using the following formula: cell population absolute count test volume = (events in cell population/events in absolute count bead region)*(beads of each test/ test volume).

Table S2: the changes of immune cells between VD and CD group in different ages

Age (years)	Test of immune cells	VD		CD		P
		N	Values	N	Values	
<=1	CD3+	285	4296.43 ±2404.72	285	4210.04 ±2575.82	0.68
	CD3+%	285	61.92 ±10	285	63.22 ±10.47	0.13
	CD3+CD4+	285	2709.55 ±1448.23	285	2730.23 ±1229.15	0.85
	CD3+CD4+%	285	39.44 ±9.99	285	42.32 ±10.4	<0.01
	CD3+CD8+	285	1411.55 ±1120.13	285	1311.94 ±1795.2	0.43
	CD3+CD8+%	285	19.88 ±7.64	285	18.31 ±7.48	0.01
	CD4/CD8	285	2.35 ±1.24	285	2.78 ±1.71	<0.01
	CD3+/HLA-DR+	222	413.62 ±565.25	225	468.7 ±1662.53	0.64
	CD3+/HLA-DR+%	222	5.9 ±7.23	225	5.85 ±9.33	0.95
	Treg	228	185.82 ±109.66	229	187.03 ±94	0.9
	Treg%	228	2.78 ±1.14	229	5.89 ±27.41	0.09
	CD3-/CD19+	285	1655.2 ±1031.89	285	1490.67 ±909.17	0.04
	CD3-/CD19+%	285	23.98 ±9.71	285	23.25 ±9.8	0.37
	CD45+	285	6954.4 ±3741.5	285	6591.21 ±3476.99	0.23
	NK	285	905.76 ±843.73	285	807.77 ±590.27	0.11
	NK%	285	12.7 ±7.54	285	12.26 ±6.34	0.45
	CD14+/HLA-DR+	223	1053.99 ±1045.35	226	1011.53 ±683.16	0.61
	CD14+/HLA-DR+%	223	86.61 ±56.5	226	82.43 ±18.45	0.29
1~2	CD3+	149	3525.06 ±2122.82	132	3445.65 ±2800.43	0.79
	CD3+%	149	60.81 ±11.82	132	59.02 ±12.79	0.23
	CD3+CD4+	149	2029.22 ±1230.33	132	1777.88 ±1159.99	0.08
	CD3+CD4+%	149	35.29 ±10.04	132	32.98 ±9.93	0.05
	CD3+CD8+	149	1230.47 ±1028.16	132	1423.72 ±1818.7	0.28
	CD3+CD8+%	149	20.85 ±8.45	132	21.91 ±10.44	0.35
	CD4/CD8	149	1.99 ±1.05	132	1.85 ±1	0.25
	CD3+/HLA-DR+	118	397.32 ±810.58	107	479.97 ±1055.61	0.51
	CD3+/HLA-DR+%	118	6.13 ±6.67	107	6.4 ±7.5	0.77
	Treg	120	135.71 ±93.99	108	128.54 ±91.54	0.56
	Treg%	120	2.44 ±1.09	108	2.4 ±1.2	0.81
	CD3-/CD19+	149	1416.43 ±912.58	132	1430.22 ±992.82	0.9
	CD3-/CD19+%	149	25.16 ±10.3	132	27.92 ±12.95	0.05
	CD45+	149	5728.41 ±3083.25	132	5456.22 ±3653.48	0.5
	NK	149	712.89 ±565.06	132	613.07 ±475.27	0.11
	NK%	149	12.8 ±9.32	132	11.42 ±6.1	0.14
	CD14+/HLA-DR+	119	953.16 ±618.03	107	867.5 ±619.05	0.3
	CD14+/HLA-DR+%	119	84.08 ±18.92	107	87.49 ±40.73	0.43
2~3	CD3+	111	3447.72 ±3476.26	86	3440.98 ±2729.59	0.99
	CD3+%	111	63.6 ±12.07	86	62.72 ±12.37	0.62
	CD3+CD4+	111	1546.62 ±795.08	86	1620.31 ±1019.01	0.58
	CD3+CD4+%	111	33.38 ±10.47	86	32.57 ±9.61	0.58

	CD3+CD8+	111	1618.94 ±3026.34	86	1512.16 ±2110.83	0.77
	CD3+CD8+%	111	24.65 ±13.66	86	23.96 ±13.6	0.73
	CD4/CD8	111	1.73 ±0.95	86	1.7 ±0.86	0.82
	CD3+/HLA-DR+	89	936.05 ±3058.31	74	690.63 ±1718.83	0.52
	CD3+/HLA-DR+%	89	8.55 ±13.71	74	9.32 ±13.86	0.72
	Treg	93	115.27 ±96.84	75	127.31 ±114.42	0.47
	Treg%	93	7.95 ±53.37	75	2.47 ±1.02	0.32
	CD3-/CD19+	111	1064.12 ±703.01	86	1052.63 ±654.04	0.91
	CD3-/CD19+%	111	23.84 ±10.98	86	23.44 ±11.22	0.8
	CD45+	111	5173.76 ±4122.84	86	5177.58 ±3266.03	0.99
	NK	111	587.26 ±558.46	86	614.21 ±500.53	0.72
	NK%	111	11.37 ±6.6	86	12.46 ±7.49	0.29
	CD14+/HLA-DR+	89	836.35 ±585.37	74	838.89 ±552.77	0.98
	CD14+/HLA-DR+%	89	86.92 ±15.21	74	86.56 ±15.8	0.88
3~4	CD3+	114	3218.33 ±4375.94	106	2464.69 ±2354.24	0.11
	CD3+%	114	65.57 ±10.45	106	62.04 ±11.63	0.02
	CD3+CD4+	114	1308.25 ±657.97	106	1140.97 ±676.21	0.06
	CD3+CD4+%	114	33.33 ±9.99	106	32.23 ±8.65	0.39
	CD3+CD8+	114	1640.97 ±3843.35	106	1098.22 ±1865.17	0.18
	CD3+CD8+%	114	26.19 ±13.69	106	23.55 ±12.04	0.13
	CD4/CD8	114	1.56 ±0.71	106	1.64 ±0.73	0.39
	CD3+/HLA-DR+	97	763.32 ±3063.82	91	601.82 ±2006.44	0.67
	CD3+/HLA-DR+%	97	15.32 ±68.77	91	8.45 ±14.89	0.33
	Treg	101	93.34 ±66.48	92	77.24 ±47.5	0.05
	Treg%	101	2.42 ±1	92	2.42 ±1.06	0.98
	CD3-/CD19+	114	856.51 ±494.55	106	797.48 ±473.68	0.37
	CD3-/CD19+%	114	23.05 ±9.9	106	24.54 ±10.08	0.27
	CD45+	114	4563.76 ±4843.72	106	3734.57 ±2726.58	0.12
	NK	114	438.22 ±457.16	106	423.17 ±331.92	0.78
	NK%	114	10.14 ±5.94	106	12.04 ±7.64	0.04
	CD14+/HLA-DR+	97	828.09 ±665.23	91	753.24 ±453.17	0.37
	CD14+/HLA-DR+%	97	87.91 ±15.05	91	87.35 ±14.57	0.8
4~5	CD3+	94	2480.99 ±2344.52	54	2690.52 ±2676.08	0.63
	CD3+%	94	65.44 ±12.7	54	66.01 ±10.36	0.77
	CD3+CD4+	94	1098.02 ±610.15	54	1183.16 ±676.25	0.45
	CD3+CD4+%	94	33.13 ±9.58	54	32.77 ±7.47	0.8
	CD3+CD8+	94	1144.09 ±1900.14	54	1191.64 ±1865.78	0.88
	CD3+CD8+%	94	25.35 ±12.12	54	25.8 ±10.53	0.82
	CD4/CD8	94	1.56 ±0.92	54	1.45 ±0.57	0.37
	CD3+/HLA-DR+	79	448.26 ±1457.47	48	855.83 ±2602.05	0.32
	CD3+/HLA-DR+%	79	7.37 ±12.42	48	10.72 ±15.49	0.21
	Treg	80	72.5 ±44.96	49	113.65 ±137.57	0.05
	Treg%	80	2.3 ±1.32	49	2.53 ±0.91	0.25
	CD3-/CD19+	94	662.37 ±401.72	54	668.76 ±374.7	0.92

	CD3-/CD19+%	94	22.11 ±11	54	20.86 ±9.21	0.46
	CD45+	94	3553.38 ±2506.96	54	3830.01 ±3062.16	0.57
	NK	94	372.82 ±291.1	54	416.54 ±370.97	0.46
	NK%	94	11.32 ±7.14	54	11.67 ±7.61	0.78
	CD14+/HLA-DR+	77	1349.43 ±5500.39	48	666.79 ±462.39	0.28
	CD14+/HLA-DR+%	77	86.42 ±14.87	48	82.02 ±17.83	0.16
5~6	CD3+	32	2841.21 ±3074.45	30	2308.98 ±2299.4	0.44
	CD3+%	32	67.92 ±11.29	30	63.67 ±10.54	0.13
	CD3+CD4+	32	1044.18 ±575.18	30	1051.5 ±777.02	0.97
	CD3+CD4+%	32	30.47 ±9.66	30	32.88 ±10.35	0.35
	CD3+CD8+	32	1544.19 ±2633.29	30	1046.16 ±1942.18	0.4
	CD3+CD8+%	32	30.27 ±14.39	30	24.22 ±12.2	0.08
	CD4/CD8	32	1.18 ±0.51	30	1.57 ±0.67	0.01
	CD3+/HLA-DR+	29	700.23 ±2021.58	25	538.51 ±2002.1	0.77
	CD3+/HLA-DR+%	29	8.98 ±13.96	25	7.42 ±14.68	0.69
	Treg	29	76.98 ±51.33	26	93.29 ±98.03	0.45
	Treg%	29	2.31 ±0.89	26	2.82 ±2.75	0.37
	CD3-/CD19+	32	562.64 ±284.71	30	677.96 ±515.16	0.29
	CD3-/CD19+%	32	18.15 ±7.28	30	21.69 ±9.15	0.1
	CD45+	32	3856.43 ±3346.97	30	3442.91 ±2671.42	0.59
	NK	32	410.08 ±295.37	30	399.12 ±353.29	0.9
	NK%	32	12.66 ±7.22	30	13.17 ±8.7	0.81
	CD14+/HLA-DR+	29	660.56 ±462.05	25	584.24 ±421.32	0.53
	CD14+/HLA-DR+%	29	84.33 ±17.51	25	79.26 ±22.24	0.36

CD: caesarean delivery, VD: vaginal delivery, N: Number of participants; CD3+: The number of CD3+ T cells/μl; CD3+%: The ratio of CD3+ T cell in total lymphocyte; CD3+CD4+: The number of CD3+CD4+ T cells/μl; CD3+CD4+%: The ratio of CD3+CD4+ T cell in total lymphocyte; CD3+CD8+: The number of CD3+CD8+ T cells/μl; CD3+CD8+%: The ratio of CD3+CD8+ T cell in total lymphocyte; CD4/CD8: the ratio of CD4+ T cell versus CD8+ T cell; CD3+/HLA-DR+: The number of CD3+/HLA-DR+ T cells/μl; CD3+/HLA-DR+%: The ratio of CD3+/HLA-DR+ T cell in total lymphocyte; Treg: The number of regulatory T cells/μl; Treg%: The ratio of regulatory T cell in total lymphocyte; CD3-/CD19+: The number of B cells/μl; CD3-/CD19+%: The ratio of B cell in total lymphocyte; CD45+: The number of CD45+ cells/μl; NK: The number of NK cells/μl; NK%: The ratio of NK cell in total lymphocyte; CD14+/HLA-DR+: The number of activated monocytes cells/μl; CD14+/HLA-DR+%: The ratio of activated monocytes in total monocytes.

All tests of these cells are done by flow cytometry. The absolute number (cells/μL) of positive cells in the sample can be determined by comparing cellular events to bead events. Absolute counts of cells are calculated by BD FACSCanto clinical software using the following formula: cell population absolute count test volume = (events in cell population/events in absolute count bead region)*(beads of each test/ test volume).
